# Supplementary material for: Productivity growth, economies of scale and scope in the water and sewerage industry: The Chilean case
Source: PLoS One. 2021 May 28;16(5):e0251874. doi: 10.1371/journal.pone.0251874 (PMC8162666; doi:10.1371/journal.pone.0251874)
Supplement: S1 Table — (DOCX) [file pone.0251874.s001.docx]

| Variables | Coeff. | St. Error | t-stat | p-value |
| --- | --- | --- | --- | --- |
| Firm specific dummy 1 | 0.476 | 0.120 | 3.970 | **0.000** |
| Firm specific dummy 2 | 0.731 | 0.114 | 6.428 | **0.000** |
| Firm specific dummy 3 | 0.606 | 0.159 | 3.805 | **0.000** |
| Firm specific dummy 4 | 0.613 | 0.173 | 3.551 | **0.000** |
| Firm specific dummy 5 | 0.518 | 0.159 | 3.257 | **0.000** |
| Firm specific dummy 6 | 0.564 | 0.159 | 3.539 | **0.000** |
| Firm specific dummy 7 | -0.157 | 0.065 | -2.407 | **0.017** |
| Firm specific dummy 8 | 1.131 | 0.160 | 7.074 | **0.000** |
| Firm specific dummy 9 | 0.347 | 0.075 | 4.625 | **0.000** |
| Firm specific dummy 10 | 0.363 | 0.099 | 3.653 | **0.000** |
| Firm specific dummy 11 | 0.356 | 0.068 | 5.249 | **0.000** |
| Firm specific dummy 12 | -0.536 | 0.129 | -4.171 | **0.000** |
| Firm specific dummy 13 | -0.925 | 0.203 | -4.562 | **0.000** |
| Firm specific dummy 14 | -0.428 | 0.191 | -2.241 | **0.026** |
| Firm specific dummy 15 | 0.428 | 0.109 | 3.931 | **0.000** |
| Firm specific dummy 16 | 0.664 | 0.101 | 6.571 | **0.000** |
| Firm specific dummy 17 | 0.809 | 0.136 | 5.936 | **0.000** |
| Firm specific dummy 18 | 0.906 | 0.154 | 5.882 | **0.000** |
| Firm specific dummy 19 | 0.907 | 0.138 | 6.579 | **0.000** |

S1 Table. Estimated results from firm-specific dummies
