# Supplementary material for: Productivity growth, economies of scale and scope in the water and sewerage industry: The Chilean case
Source: PLoS One. 2021 May 28;16(5):e0251874. doi: 10.1371/journal.pone.0251874 (PMC8162666; doi:10.1371/journal.pone.0251874)
Supplement: Attachment — Submitted filename: Response to Reviewers.docx [file pone.0251874.s002.docx]

**General response to the editor and reviewers:**

The authors would like to thank the editor and the reviewers for considering this manuscript and for their insightful feedback. A number of comments and questions from the reviewers were made which have been carefully answered and clarified in the revision notes provided. The comments have been carefully addressed in the manuscript. The revised manuscript is now improved, and the revision notes contain detailed answers to each reviewer’s comments and questions. Our responses to each one of them are presented below.

**Comments to the Author**:

**Reviewer #1:**

Molinos-Senante and Maziotis present a case study for Chilean water utilities in a very good manner. I must also say that I have been following their work for a while and I think Maria is doing great work on water utilities. The manuscript describes the use of a quadratic function for costs using actual data. Conclusions would have an impact on public policy, and results and methods are sound. However, I have a couple of comments regarding the manuscript structure and some topics that to discuss more profoundly:

I. The Introduction gives some background regarding economies of scale and scope. I think the authors should also discuss how both concepts are related to regulation and water supplies. Moreover, authors could discuss here, in another piece, how climate and climate change affect pricing. I would also suggest introducing and discussing whether the Chilean pricing procedures (against the "model company") and parametrization affect cost functions.

**Response:** Thank you for your insightful comment. The following text has been added to the introduction to discuss how economies of scale and scope are linked with water regulation. We also added more text regarding the Chilean water regulation, pricing and climate change:

*“The concept of economies of scale is of great importance for the water regulator and regulated companies for the following reasons. An industry that operates under increasing economies of scale suggests that further adjustments in companies’ scale of operations could lead to lower costs. This means that the regulator could promote mergers among companies as cost savings could be achieved if companies become bigger. In contrast, if the industry operates under decreasing economies of scale, then further increases in the size of the companies could not be justified from a cost perspective. This implies that the regulator should not promote policies that merge companies as overall costs would not go down. The concept of economies of scope also receives considerable attention by water regulator and regulated companies. The existence of positive economies of scope between water and sewerage services suggests that the joint provision of these services brings substantial cost savings. This means that water and sewerage companies should remain integrated, and the regulator should not promote policies that could unbundle these services. Breaking up the water and sewerage companies into smaller companies that focus on the provision of water services only and on the supply of sewerage services only might not lower production costs. By contrast, negative economies of scope imply that cost savings could be achieved if the water and sewerage services are provided by separate utilities. Understanding therefore the cost structure of the industry could help policy makers to deliver the water and wastewater services in an efficient and affordable manner. Therefore, the regulator and regulated companies have a significant role to play. For instance, the regulator should protect the customers from the monopoly abuse of the water companies. Moreover, it should monitor the performance of the companies by ensuring that any costs savings are passed to the customers in terms of lower prices. Finally, the regulator should ensure that sufficient finance is available to the water companies to operate and upgrade the network so that enough water is available for present and future generations”.*

*“The monopolistic nature of the Chilean water companies required the establishment of a national regulator, the Superintendencia de Servicios Sanitarios (SISS). Its duties are to ensure the financial sustainability of the sector, set tariffs and keep customers’ bills at an affordable level, enhance economic efficiency and water conservation (Molinos-Senante, 2018). Tariffs are set based on the definition of a hypothetical efficient water company (a “model company”) (Molinos-Senante et al., 2019). They need to cover operating and capital expenditure, taxes and guarantee a fair return to capital invested.* *Water companies need to ensure that sufficient water is available for people which might be difficult in the framework of climate change. To achieve this objective, water companies should buy water rights from other water users such as agriculture or mining at market price. Given the model applied in Chile to set water tariffs, the purchase of water rights by the water companies might impact on water tariffs. However, detailed studies by Donoso (2015) and SISS (2015) evidenced that the scarcity value of water, i.e., the price of water rights, is not reflected in the Chilean water tariffs”.*

Donoso, G. (2015). Water Pricing in Chile. In: A. Dinar, V. Pochat, & J. Albiac (Eds.), Water Pricing Experiences and Innovations, Vol. 9, pp. 471. Switzerland: Springer-Verlag.

SISS (Superintendencia de Servicios Sanitarios) (2015). Management report of Chilean water and sewerage companies. Available at: https://www.siss.gob.cl/586/w3-propertyvalue-6415.html

II. Lines 101-128 are more a discussion (or literature review) than a methodological description. I would like to suggest a critical appraisal of the quadratic function as compared to other approaches (just a couple of lines touched this issue).

**Response:** Thank you for your insightful comment. We would like to note that in those line we do offer a critical appraisal of the quadratic cost function over other functional forms. For instance, the manuscript already states that “Compared to translog function, a quadratic cost function takes into account zero values. Other functional forms e.g., composite or generalized composite are difficult to estimate and converge and in other cases their coefficients do not have a direct economic meaning (Triebs et al., 2016)”. We also note the substantial advantage of quadratic cost function over other functional forms such as translog, composite: “it allowed us to quantify in monetary terms the savings from the joint or separate production of multi-output services”. We also highlight some limitations of the quadratic cost function: “Limitations of the quadratic cost function involve the imposition of homogeneity in input prices as a regularity condition which may sacrifice flexibility (Caves et al., 1980).” We also demonstrate how these limitations could be overcome: “However, this issue can be dealt with by normalizing cost and input prices by one of the input prices prior to estimation (Martınez-Budrıa et al., 2003; Molinos-Senante and Maziotis, 2020).”

III. To help readiness, I suggest adding a table of variables. Also, please provide more details regarding the statistical significance test.

**Response**: Thank you for your insightful comment. We provide below the list of the specific dummies variables. To avoid increasing the length of the main text, the following table has been included as supplementary material:

Table S1. Estimated results from firm-specific dummies

| Variables | Coeff. | St.Error | t-stat | p-value |
| --- | --- | --- | --- | --- |
| Firm specific dummy 1 | 0.476 | 0.120 | 3.970 | **0.000** |
| Firm specific dummy 2 | 0.731 | 0.114 | 6.428 | **0.000** |
| Firm specific dummy 3 | 0.606 | 0.159 | 3.805 | **0.000** |
| Firm specific dummy 4 | 0.613 | 0.173 | 3.551 | **0.000** |
| Firm specific dummy 5 | 0.518 | 0.159 | 3.257 | **0.000** |
| Firm specific dummy 6 | 0.564 | 0.159 | 3.539 | **0.000** |
| Firm specific dummy 7 | -0.157 | 0.065 | -2.407 | **0.017** |
| Firm specific dummy 8 | 1.131 | 0.160 | 7.074 | **0.000** |
| Firm specific dummy 9 | 0.347 | 0.075 | 4.625 | **0.000** |
| Firm specific dummy 10 | 0.363 | 0.099 | 3.653 | **0.000** |
| Firm specific dummy 11 | 0.356 | 0.068 | 5.249 | **0.000** |
| Firm specific dummy 12 | -0.536 | 0.129 | -4.171 | **0.000** |
| Firm specific dummy 13 | -0.925 | 0.203 | -4.562 | **0.000** |
| Firm specific dummy 14 | -0.428 | 0.191 | -2.241 | **0.026** |
| Firm specific dummy 15 | 0.428 | 0.109 | 3.931 | **0.000** |
| Firm specific dummy 16 | 0.664 | 0.101 | 6.571 | **0.000** |
| Firm specific dummy 17 | 0.809 | 0.136 | 5.936 | **0.000** |
| Firm specific dummy 18 | 0.906 | 0.154 | 5.882 | **0.000** |
| Firm specific dummy 19 | 0.907 | 0.138 | 6.579 | **0.000** |

IV. In lines 277-278 the authors report the importance of water sources. Please, briefly discuss the importance of groundwater due to availability, depth, and operational costs.

**Response**: Thank you for your insightful comment. We added the following text in the manuscript:

*“This is attributed to the fact that abstracting water from this type of water resources might require high energy which could lead to higher operational costs.* *Moreover, as water availability decreases, the depth of wells increases and therefore, higher energy requirements might be needed to pump and treat water from groundwater and mixed water resources which could have an impact on operational costs”.*

V. In lines 295-297, please elaborate on the importance of economies of scale in Chile.

**Response**: Thank you for your insightful comment. We added the following text:

*“This finding suggests that if Chilean water companies could increase the size of their operations by acquiring more customer base, it could lead to lower production costs. This means that neighbor companies could potentially merge to benefit from economies of scale.* *Currently, this option is not feasible because each water company has defined its concession area (i.e., operational territory) where it can provide water and sewerage services. However, this a policy that the regulator could potentially consider as it can be justified from a costs perspective”.*

VI. Do regulatory strength influence the economies of scale and scope? (lines 298-325).

**Response**: Thank you for your comment. There might be several factors that could influence economies of scale and scope. For instance, the allocation of resources, i.e., operating and capital expenditure, the prices of these resources, the volumes of water delivered and the number of customers receiving wastewater treatment, and several operating characteristics such as type of water resources and type of ownership. The regulatory framework is another factor that could influence the cost structure of the industry. It is a combination of several factors that could influence economies of scale and scope.

VII. What did drive TFP changes in 2010/11, 2011/12, 2014/15, and 2016/17? (lines 345-356).

**Response**: Thank you for your comment. As we noted in the manuscript, in 2010/11 and 2011/12 TFP increased due to an increase in technical change (TC). This means that the adoption of new technologies to improve network performance could have led to lower production costs. For instance, technological advancements to more accurately predict bursts or water leakages in the network could have led to lower operational costs. Another example could be the use of energy efficient technologies to pump water from different resources could have led to cost savings. As we noted in the manuscript, in 2014/15 and 2016/17 TFP increased due to the positive scale effect which offset technical regress. This means that increases in companies’ scale of operations by providing water and wastewater services to more people had led to lower production costs.

VIII. I am not a native English speaker, but there few grammar and word choices that authors should correct. (e.g. lines 268, 252).

**Response**: Thank you for your comment. We corrected any grammatical errors and typos.

IX. Please, explore other visualisation options for figures 2 and 3.

**Response**: Thank you for your comment. Figures 2 and 3 have been updated.

**Reviewer #2:**

This study beholds practical values as examining the existence of economies of scale and scope in the Chilean water and sewerage industry. The writing is fluent, plain, and readable. However, in my view, there still exist some places for improvement:
First of all, certain aspects of contents in this study need to be expanded to increase its theoretical contributions. As the introduction part lacks address the value (or the aim) of examining the existence of economies of scale and scope: will confirming this existence help to industry regulation, industry investment, or enhancement of citizen’s well-being? And how? I do not see adequate background knowledge on that.

**Response**: Thank you for your insightful comment. We agree with you so we added the following text in the introduction:

*“The concept of economies of scale is of great importance for the water regulator and regulated companies for the following reasons. An industry that operates under increasing economies of scale suggests that further adjustments in companies’ scale of operations could lead to lower costs. This means that the regulator could promote mergers among companies as cost savings could be achieved if companies become bigger. In contrast, if the industry operates under decreasing economies of scale, then further increases in the size of the companies could not be justified from a cost perspective. This implies that the regulator should not promote policies that merge companies as overall costs would not go down. The concept of economies of scope also receives considerable attention by regulator and regulated companies. The existence of positive economies of scope between water and sewerage services suggests that the joint provision of these services brings substantial cost savings. This means that water and sewerage companies should remain integrated, and the regulator should not promote policies that could unbundle these services. Breaking up the water and sewerage companies into smaller companies that focus on the provision of water services only and on the supply of sewerage services only might not lower production costs. By contrast, negative economies of scope imply that cost savings could be achieved if the water and sewerage services are provided by separate utilities. Understanding therefore the cost structure of the industry could help policy makers to deliver the water and wastewater services in an efficient and affordable manner. Therefore, the water regulator and regulated companies have a significant role to play here. For instance, the regulator should protect the customers from the monopoly abuse of the water companies. Moreover, it should monitor the performance of the companies by ensuring that any costs savings are passed to the customers in terms of lower prices. Finally, the regulator should ensure that sufficient finance is available to the water companies to operate and upgrade the network so that enough water is available for present and future generations”.*

*“The monopolistic nature of the Chilean water companies required the establishment of a national regulator, the Superintendencia de Servicios Sanitarios (SISS). Its duties are to ensure the financial sustainability of the sector, set tariffs and keep customers’ bills at an affordable level, enhance economic efficiency and water conservation (Molinos-Senante, 2018). Tariffs are set based on the definition of a hypothetical efficient water company (a “model company”) (Molinos-Senante et al., 2019). They need to cover operating and capital expenditure, taxes and guarantee a fair return to capital invested. Water companies need to ensure that sufficient water is available for people which might be difficult in the framework of climate change. To achieve this objective, water companies should buy water rights from other water users such as agriculture or mining at market price. Given the model applied in Chile to set water tariffs, the purchase of water rights by the water companies might impact on water tariffs. However, detailed studies by Donoso (2015) and SISS (2015) evidenced that the scarcity value of water, i.e., the price of water rights, is not reflected in the Chilean water tariffs”.*

Donoso, G. (2015). Water Pricing in Chile. In: A. Dinar, V. Pochat, & J. Albiac (Eds.), Water Pricing Experiences and Innovations, Vol. 9, pp. 471. Switzerland: Springer-Verlag.

SISS (Superintendencia de Servicios Sanitarios) (2015). Management report of Chilean water and sewerage companies. Available at: https://www.siss.gob.cl/586/w3-propertyvalue-6415.html

For another thing, focusing on directly economies of scale in the Chilean water and sewerage industry could easily lose reading interests from international readers out of Chile. Thus this study should offer more content under the theme as “What can other countries learn from Chile’s case.” I see such attempts in Section 4: the attempts were good; however, those discussions were not conducted in-depth. For instance, from Line 304, Page 16 to Line 325, Page 17, the authors compared their findings with previous studies in some other countries; however, the comparison were limited to simply the results (existence of economies of scale or not) and lacked at providing some possible explanations to that divergence in results. Although the authors quoted Saal et al. (2013) and Molinos-Senante et al. (2017) acclaiming that the result divergence was caused by the methodology used, I am more curious about: (1) What is the authors’ opinion about this divergence in results? Do you believe in the methodology-caused explanation? (2) If yes, considering the capture of economies of scale is method-specific, can you offer some more comments on the previous methods, conduct comparisons among those methods, and then acclaim the advantage of the current method in capturing economies of scale based on the results? (3) Aside from the methodology-caused explanation, are there any other explanations for the result divergence? More specifically, as is mentioned in the introduction part, this study is among the earliest studies examining economies of scale and scope in developing countries; thus, could more explanations be offered from the perspective of the developing/developed country difference?

**Response**: Thank you for your insightful comment. First, we want to elaborate on the existence of economies of scale in the Chilean water industry based on our results. Therefore, we added the following text in the manuscript:

*“This finding suggests that if Chilean water companies could increase the size of their operations by acquiring more customer base, it could lead to lower production costs. This means that neighbor companies could potentially merge to benefit from economies of scale. Currently, this option is not feasible because each water company has defined its concession area (i.e., operational territory) where it can provide water and sewerage services. However, this a policy that the regulator could potentially consider as it can be justified from a costs perspective”.*

Then we make the following comments with respect to economies of scope. Moreover, we would like to note that there has not been any other published studies in the Chilean water industry which estimated economies of scope between water and sewerage services. Therefore, we wanted to see how our findings are corroborated with those obtained from other water industries worldwide. For instance, De Witte and Marques (2011), Bottasso et al. (2011), Stone and Webster Consultants (2004) found diseconomies of scope between water and sewerage services. This finding is corroborated with ours. Other studies by Saal and Parker (2000), Molinos-Senante and Maziotis (2017; 2018) found the opposite. Thus, there might be some convergence or divergence in findings with past studies. This is very rational as the aforementioned studies conducted in countries other than Chile. This means that costs (e.g., operating and capital expenditure) in those industries are completely different from those of Chile. This can be considered the most important reason why results converge or diverge. Moreover, another reason that results of our study might differ from past studies is because of the methodology used. For instance, De Witte and Marques (2011) used non-parametric techniques to find diseconomies of scope whereas our study used a parametric approach to reach the same conclusion. Another example is the choice of functional form. For instance, Bottasso et al. (2011) used a composite cost function and found diseconomies of scope between water and sewerage services in England and Wales. Other studies by Saal and Parker (2000) and Molinos-Senante and Maziotis (2017; 2018) used translog and quadratic cost functions to report economies of scope between water and sewerage services in England and Wales. We would like to note that all past studies used robust methodologies to study the concept of economies of scale and scope and all cited papers were peer-reviewed. Each methodology has its own assumptions and any divergence or convergence in results with past studies depends on the method, costs (a substantial factor), inputs, outputs, environmental variables, sample of companies, time period. There is not one “correct” method to estimate economies of scale and scope. We believe that the researcher should have a good understanding of the industry and its environment before estimating productivity change or economies of scale and scope. The choice of method, inputs and outputs is based on her/his research objective. Results should be justified based on the above factors (e.g. method, costs, outputs etc) and any divergence or convergence with past studies should be explained by those factors. Our study provided the first step to examine the concept of economies of scope in developing countries. We shed more light on the existence of economies of scope between water and sewerage services from a developing country perspective. More evidence is definitely required to understand the cost structure of this industry. We are committed to work towards this objective.

As for the methodology, considering the model this study relied on upon to estimate is very classic, sensitivity analysis and heterogeneity analysis should be considered to add the estimation validity and offer more research implications.

**Response**: Thank you very much for your insightful comment. We controlled for heterogeneity by including firm-specific dummies and environmental variables. The former captures managerial inability and the latter captures the impact of operating characteristics such as customer density, ownership on total costs. In the supplementary material we report the firm-specific dummies and their statistically significance. We don’t include them in Table 2 for spacious reasons. Moreover, in Table 2 the estimated coefficients of outputs and input price are positive and statistically significant from zero. This means that as output and input price increase then total costs increase as well, which satisfies the property of a cost function. This finding reinforces the robustness of our method.

Table S1. Estimated results from firm-specific dummies

| Variables | Coeff. | St.Error | t-stat | p-value |
| --- | --- | --- | --- | --- |
| Firm specific dummy 1 | 0.476 | 0.120 | 3.970 | **0.000** |
| Firm specific dummy 2 | 0.731 | 0.114 | 6.428 | **0.000** |
| Firm specific dummy 3 | 0.606 | 0.159 | 3.805 | **0.000** |
| Firm specific dummy 4 | 0.613 | 0.173 | 3.551 | **0.000** |
| Firm specific dummy 5 | 0.518 | 0.159 | 3.257 | **0.000** |
| Firm specific dummy 6 | 0.564 | 0.159 | 3.539 | **0.000** |
| Firm specific dummy 7 | -0.157 | 0.065 | -2.407 | **0.017** |
| Firm specific dummy 8 | 1.131 | 0.160 | 7.074 | **0.000** |
| Firm specific dummy 9 | 0.347 | 0.075 | 4.625 | **0.000** |
| Firm specific dummy 10 | 0.363 | 0.099 | 3.653 | **0.000** |
| Firm specific dummy 11 | 0.356 | 0.068 | 5.249 | **0.000** |
| Firm specific dummy 12 | -0.536 | 0.129 | -4.171 | **0.000** |
| Firm specific dummy 13 | -0.925 | 0.203 | -4.562 | **0.000** |
| Firm specific dummy 14 | -0.428 | 0.191 | -2.241 | **0.026** |
| Firm specific dummy 15 | 0.428 | 0.109 | 3.931 | **0.000** |
| Firm specific dummy 16 | 0.664 | 0.101 | 6.571 | **0.000** |
| Firm specific dummy 17 | 0.809 | 0.136 | 5.936 | **0.000** |
| Firm specific dummy 18 | 0.906 | 0.154 | 5.882 | **0.000** |
| Firm specific dummy 19 | 0.907 | 0.138 | 6.579 | **0.000** |

Besides, I noticed that data was collected for the period 2010-2017 (which a relatively small-size sample); the authors should address why data before that time range is not accessible and write it into the research limitation.

**Response**: Thank you for your insightful comment. We focus on this period because of data limitations. We added the following sentence in the conclusion section:

*“Our research does not come without limitations. The study period involved was considerably small as it covered the period 2010-17. Unfortunately, due to the lack of available data for several variables such as capital expenditure we could not extend the dataset before 2010. More efforts in the future will focus on including more recent data on inputs, outputs and environmental variables and if possible collect data on additional variables that could impact water companies’ performance such as volumes of sewerage collected and treated, drinking water and sewerage treatment quality”.*

Finally, more details about the case of Chile’s water and sewerage industry system and development are expected to be offered to readers as a research background. Correspondingly, practical implications in the conclusion part (such as content from Line 430, Page 21 to the end) should be discussed more related to Chile’s situations.

**Response**: Thank you for your insightful comment. We added the following text in the introduction:

*“The monopolistic nature of the Chilean water companies required the establishment of a national regulator, the Superintendencia de Servicios Sanitarios (SISS). Its duties are to ensure the financial sustainability of the sector, set tariffs and keep customers’ bills at an affordable level, enhance economic efficiency and water conservation (Molinos-Senante, 2018). Tariffs are set based on the definition of a hypothetical efficient water company (a “model company”) (Molinos-Senante et al., 2019). They need to cover operating and capital expenditure, taxes and guarantee a fair return to capital invested. Water companies need to ensure that sufficient water is available for people which might be difficult in the framework of climate change. To achieve this objective, water companies should buy water rights from other water users such as agriculture or mining at market price. Given the model applied in Chile to set water tariffs, the purchase of water rights by the water companies might impact on water tariffs. However, detailed studies by Donoso (2015) and SISS (2015) evidenced that the scarcity value of water, i.e., the price of water rights, is not reflected in the Chilean water tariffs”.*

Donoso, G. (2015). Water Pricing in Chile. In: A. Dinar, V. Pochat, & J. Albiac (Eds.), Water Pricing Experiences and Innovations, Vol. 9, pp. 471. Switzerland: Springer-Verlag.

SISS (Superintendencia de Servicios Sanitarios) (2015). Management report of Chilean water and sewerage companies. Available at: https://www.siss.gob.cl/586/w3-propertyvalue-6415.html

We also added the following text to the conclusion section:

*“More particularly, for the case of the Chilean water industry the findings could have several interesting implications. The fact that the water industry operates under increasing economies of scale means that the regulator could consider mergers among companies as a policy that could potentially lead to lower costs. Moreover, having smaller companies that offer water services only or sewerage services only could lead to lower costs. The use of new technologies could allow companies to run their business more efficiently for instance by reducing water leakage in pipes or by using energy efficiency techniques to abstract water from different resources. This could potentially lead to higher levels of efficiency and productivity growth. Subsequently, any cost savings could pass to customers in terms of lower tariffs. An efficient operation of water and wastewater business would boost environmental sustainability contributing positively to people’s health and well-being”.*
